# Evaluating indices of insulin resistance and estimating the prevalence of insulin resistance in a large biobank cohort

**DOI:** 10.3389/fendo.2025.1591677

**Published:** 2025-05-12

**Authors:** Usama Aliyu, Salman M. Toor, Ibrahem Abdalhakam, Mohamed A. Elrayess, Abdul Badi Abou−Samra, Omar M. E. Albagha

**Affiliations:** ^1^ College of Health and Life Sciences (CHLS), Hamad Bin Khalifa University (HBKU), Qatar Foundation (QF), Doha, Qatar; ^2^ Qatar Metabolic Institute, Hamad Medical Corporation (HMC), Doha, Qatar; ^3^ Biomedical Research Center, QU Health, Qatar University, Doha, Qatar; ^4^ College of Medicine, QU Health, Qatar University, Doha, Qatar; ^5^ Centre for Genomic and Experimental Medicine, Institute of Genetics and Cancer, University of Edinburgh, Edinburgh, United Kingdom

**Keywords:** insulin resistance, type 2 diabetes, HOMA-IR, TyG index, population study

## Abstract

**Introduction:**

Insulin resistance (IR) is involved in the pathogenesis of various metabolic disorders. Several surrogate indices of IR have been proposed. We assessed the performance of seven clinically relevant indirect measures of IR and estimated the prevalence of IR in a large population-based cohort.

**Methods:**

The study was conducted on fasting individuals from the Qatar biobank (QBB) participants (*n* = 7,875). Individuals were considered insulin sensitive (IS) if lean, not diagnosed with diabetes, no hypertriglyceridemia, and not on lipid-lowering drugs, while individuals with Type 2 diabetes (T2D) were considered insulin resistant (IR). Cut-offs were determined as the top or lowest quartile values in the IS participants. The performance of IR indices was based on area under the curve (AUC), sensitivity and specificity.

**Results:**

The cut-off for HOMA-IR was determined at 1.878, HOMA2-IR (insulin); 1.128, HOMA2-IR (C-peptide); 1.307, QUICKI; 0.347, TyG; 8.281, McAi; 7.727 and 1.718 for TG/HDL. All IR indices analyzed yielded AUC values ranging from 0.83 to 0.92. TyG was the most robust measure for IR (AUC = 0.92, Sensitivity = 0.90, Specificity = 0.79). The overall prevalence of IR in Qatar was estimated at ~51 – 65%.

**Conclusions:**

TyG index was the most robust index for determining IR in the Qatari population. The proposed cut-offs could serve as a reference in Middle Eastern populations for IR screening.

## 1 Introduction

The rising global prevalence of metabolic disorders poses an alarming global health concern (1). Metabolic disorders refer to a spectrum of metabolic dysregulations, which include hypertension (HTN), Type 2 Diabetes (T2D), hyperlipidemia, obesity and metabolic dysfunction-associated steatotic liver disease (MASLD). Around 43.8 million people globally were affected by T2D, 18.5 million by HTN, and a staggering 1.2 billion by MASLD in 2019 (1). The burden of T2D has reached ~73 million individuals in the Middle East and North African (MENA) region, which is the highest regional prevalence (16.2%) in the world and accounted for the highest regional diabetes-related morbidity (24.5%) (2). Qatar has experienced an epidemic of T2D, reaching ~17% of the adult population (3), attributed predominantly to the shift from active to sedentary lifestyle and a consequent commensurate rise in obesity (4).

Metabolic disorders recurrently occur in tandem and share common risk factors. Insulin resistance (IR) has been described as the cardinal driver in the etiopathogenesis of a plethora of metabolic diseases, including Metabolic syndrome (MS), Polycystic Ovary Syndrome (PCOS), and Metabolic-Associated Fatty Liver Disease (MAFLD) (5–7). IR is defined by the reduced tissue sensitivity to insulin to adequately ignite cellular response. The gold standard for measuring IR is the Hyperinsulinemic-euglycemic clamp (HEC) (8). However, HEC is highly invasive, laborious, and unfeasible in large studies. Consequently, several low-invasive surrogate measures of IR have been proposed, broadly divided into the oral glucose tolerance test (OGTT) and fasting-based indices (9). Their utilization is supported by high correlation with the gold standard and high reproducibility (10). Some of the commonly used fasting-based IR indices include Homeostasis Model Assessment for Insulin Resistance (HOMA-IR) (11) and its improved version HOMA2-IR (12), Quantitative Insulin Sensitivity Check Index (QUICKI) (13), Triglyceride/High-Density Lipoprotein (TG/HDL) ratio (14), Triglyceride - Glucose (TyG) index (15), and McAuley index (MCAi) (16).

Studies have shown that the performances of IR indices and optimal cut-offs vary among populations and between genders (17–19). Selected studies have compared the performance of surrogate measures of IR in different populations. Endukuru et al. (2020) reported HOMA-IR (cut-off ≥ 2.86) as the most accurate metric in diagnosing metabolic syndrome (MS) among Indian adults, compared to other indices (20). In contrast, Ádány et al. (2020) and Mir et al. (2021) reported TyG as the optimal diagnostic/prognostic index for MS/IR than other indices in Hungarian (cut-off ≥ 4.69) (21) and Polish (cut-off ≥ 8.741) (18) individuals respectively. Population-based and gender-specific cut-offs for the commonly used surrogate measures of IR remain largely unexplored globally, including in the Middle East.

In this study, we assessed the performance of seven indirect indices of IR that are clinically useful and determined the combined and gender-specific cut-offs of these indices using clinical data of participants from the population-based Qatar Biobank (QBB) cohort. The indices for IR covered herein included HOMA-IR (11), HOMA2-IR (C-peptide) (12), HOMA2-IR (insulin) (12), QUICKI (13), TG/HDL (14), TyG index (15) and MCAi (16). TyG index outperformed all analyzed indices, evident from the highest diagnostic and discriminative performance. In addition, we estimated the prevalence of IR in the Qatari population. Our findings have potential clinical utilization in Qatar and in the wider Middle Eastern region, following validation in larger population-based clinical cohorts.

## 2 Materials and methods

### 2.1 Ethical statement

The study was conducted according to the guidelines of the Declaration of Helsinki and approved by the Institutional Review Boards of Qatar Biobank (QBB; Approval No. E-2019-QF-QBB-RES-ACC-0179-0104) and Hamad Bin Khalifa University, Doha, Qatar (Approval No. QBRI-IRB 2021-03-078). All participants provided written informed consent prior to participation in the study.

### 2.2 Study participants

The study was conducted on participants from QBB, a population-based prospective initiative by Qatar Foundation to promote biomedical research in Qatar and globally. QBB comprises adults (aged ≥ 18 years) who are Qatari nationals (98.2%) or long-term (≥ 15 years) residents of Qatar (1.8%). However, this study was restricted to include Qatari subjects only and long-term residents were not included. QBB covers extensive baseline social, demographic, clinical, metabolic, behavioral and phenotypic data, in addition to collecting biological samples. The QBB comprised 13,808 participants, of which 7,875 had fasting (≥ 8 hours) biochemical measurements. Analysis of indices of IR and performance assessment was carried out on participants with fasting measurements; however, the prevalence of IR was assessed on both fasting participants and the total study cohort. QBB participants were categorized as individuals with Type 2 diabetes (T2D) if they declared to have diabetes or on diabetes treatment. Newly diagnosed diabetes, if their HbA1C values were > 6.5% and/or random glucose values were > 11.1 mmol/l (> 200 mg/dL) and did not self-report as having diabetes nor were on diabetes treatment. Prediabetes, if they did not declare to have diabetes or on diabetes treatment and with HbA1C levels between 5.7% and 6.4% or their fasting glucose (≥ 8 hours) levels were between 5.6 and 6.9 mmol/L. Individuals were otherwise classified as normal (not diagnosed with diabetes) if they did not fall in any of the aforementioned categories.

### 2.3 Data collection and biochemical analyses

Clinical and biochemical data collection procedures for QBB participants have been described previously (22). Briefly, fasting glucose levels in serum were measured using the enzymatic method with GLUC3 glucose hexokinase kit (Roche, Switzerland) on a Cobas instrument (Roche). Serum C-peptide levels were measured using the sandwich electrochemiluminescence (ECLIA/sandwich) immunoassay using Elecsys C-peptide kit (Roche), while serum insulin was measured using ECLIA/Sandwich immunoassay also on Cobas instrument (Roche). HbA1c in blood was measured using turbidimetric inhibition immunoassay (TINIA) utilizing Tina-quant HbA1c Gen. 3 kit (Roche). Triglycerides were measured using the Enzymatic colorimetric method, and serum Low-Density Lipoprotein Cholesterol (LDL-C) and High-Density Lipoprotein Cholesterol (HDL-C) were measured using homogeneous enzymatic colorimetric test on Cobas instrument (Roche).

### 2.4 Calculation of indices of insulin resistance

Seven indices of IR were calculated for the participants that met the study inclusion criteria; Homeostasis Model Assessment for Insulin Resistance (HOMA-IR) (11), HOMA2-IR calculated using C-peptide (HOMA2-IR C-peptide) (12), HOMA2-IR calculated using insulin (HOMA2-IR insulin) (12), Quantitative Insulin Sensitivity Check Index (QUICKI) (13), Triglyceride/High-Density Lipoprotein (TG/HDL) ratio (14), Triglyceride - Glucose (TyG) index (15) and McAuley index (MCAi) (16). The formulae for calculating these indices are shown below:

HOMA-IR = Fasting Insulin (µU/mL) x Fasting Glucose (mmol/L)/22.5
QUICKI = 1/log Fasting Insulin (μU/mL) + log Fasting Glucose (mg/dL)
TG/HDL = Triglyceride (mg/dL)/High Density Lipoprotein (mg/dL)
TyG = ln (Fasting Triglyceride (mg/dL) x Fasting Glucose (mg/dL)/2)
MCAi = exp ((2.63–0.28 ln [Insulin (μU/mL)] − 0.31 ln [Triglyceride (mmol/L)]))

HOMA2-IR (C-peptide) and HOMA2-IR (insulin) were calculated using the HOMA-2 calculator (https://www.dtu.ox.ac.uk/homacalculator).

### 2.5 Performance assessment & statistical analyses

The data was partitioned into 70% for the discovery cohort (*n* = 5,512) and 30% for the testing cohort (*n* = 2,363) to identify the optimal cut-offs for IR indices. Individuals were considered insulin sensitive (IS) if they were lean (BMI ≤ 24.9), not diagnosed with diabetes, without hypertriglyceridemia (< 500 mg/dL), and not on lipid-lowering drugs (*n* = 960 from discovery cohort and *n* = 433 from testing cohort), while individuals were considered IR if they were diagnosed with Type 2 Diabetes (*n* = 1,158 from the discovery cohort and *n* = 491 from the testing cohort). The cut-offs were identified as values above the 75^th^ percentile in IS individuals for HOMA-IR, HOMA2-IR, TG/HDL, and TyG and below the 25^th^ percentile for QUICKI and MCAi in the discovery dataset. The identified cut-offs were then assessed in the testing dataset to determine sensitivity, specificity, and AUC for performance assessment in discriminating between IS and IR individuals, using the “cutpointr” package (23) in R. To mitigate the risk of overfitting, we employed a cross-validation approach with 100 bootstraps. Area Under the Curve (AUC) - Receiver Operator Curve (ROC) analysis was also carried out using “cutpointr” package in R (23). Gender-specific cut-offs were also identified using the same approach. To investigate the effect of medication on IR participants, we re-assessed the performance of the indices based on newly diagnosed T2D participants only (*n* = 149) as they were not on any diabetes medication.

All statistical analyses were performed using R software (version 4.3.1). Quantitative variables were expressed as Interquartile range; median (25^th^ - 75^th^ percentile). Categorical variables were expressed as a number (percentage). Shapiro-Wilk normality test was first used to assess the data distribution. Mann-Whitney test was used to compare quantitative variables, while Chi-square test was used for categorical variables. A *P*-value of < 0.05 was considered statistically significant. Spearman correlation test was used to assess correlations between variables using the *cor* function in base R. UpSetR plot from the UpSetR package (24) in R was used to illustrate the concordance of different IR indices in identifying IR. The performance of AUC of TyG index compared to other indices was evaluated using DeLong’s pairwise test.

### 2.6 Determining the prevalence of IR

The prevalence of IR was defined as the number of individuals with an IR index value above/below the given cut-off and expressed as a percentage of all QBB participants with fasting measurements (*n* = 7, 875), males (*n* = 3,223), females (*n* = 4,652), without diabetes (*n* = 4,843) and with prediabetes (*n* = 1,368). Moreover, we investigated the effect of fasting time on IR indices and calculated the prevalence of IR in all QBB participants (*n* = 13,808) without considering fasting time. The prevalence of IR was also calculated for different age groups (18-28, 29-38, 39-48, 49-58, and > 58 years).

## 3 Results

### 3.1 Study participants and clinical characteristics

**Figure 1:**
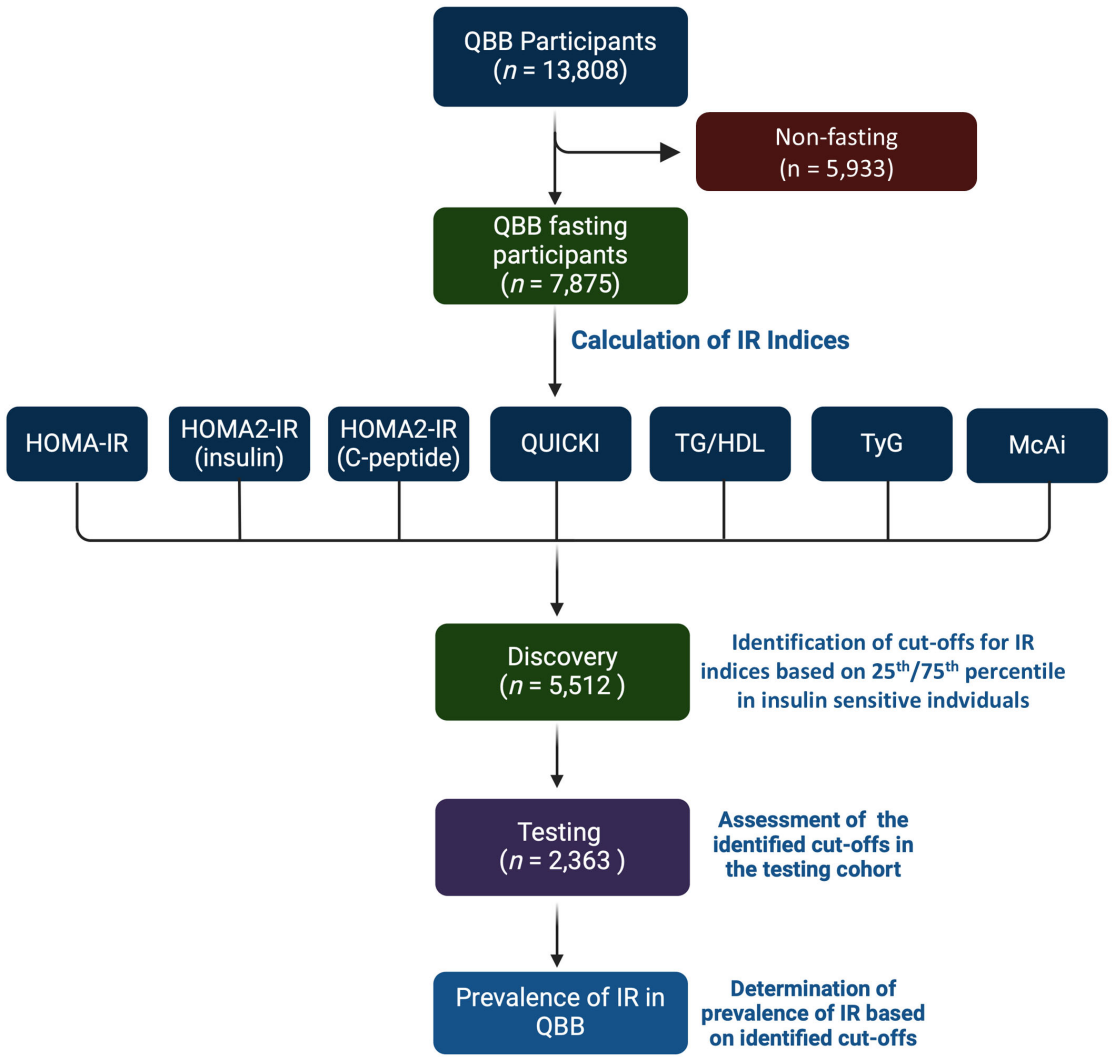
Study design. The study comprised QBB participants with clinical and fasting biochemical measurements (*n* = 7,875). Seven indices of IR were calculated for each participant. The study cohort was partitioned into 70% for discovery (*n* = 5,512) and 30% for testing cohorts (*n* = 2,363). The cut-offs were identified as values above the 75th percentile in insulin sensitive (IS) individuals for HOMA-IR, HOMA2-IR, TG/HDL, and TyG and below the 25th percentile for QUICKI and MCAi in the discovery dataset. The cut-offs were assessed in the testing dataset. Individuals that are lean (BMI ≤ 24.9), without diabetes, without hypertriglyceridemia (< 500 mg/dL), and not on lipid-lowering drugs were considered IS, and individuals with type 2 diabetes (T2D) were considered IR.

The overall study design is depicted in **Figure 1**. The clinical characteristics of the study participants and stratification into diabetes status are listed in **Table 1**. The study cohort had a median age of 39.0 and was comprised of 41.0% males and 59.0% females. Notably, around 79% of the participants had obesity or were overweight, and around 73% had a family history of diabetes from at least one parent. All variables in individuals with prediabetes and T2D showed statistically significant differences (*P*-value < 0.05) compared to individuals without diabetes.

**Table 1:** Characteristics of study cohort.

Parameter	Full cohort	Fasting cohort			
	All (*n* = 13,808)	All (*n* = 7,875)*	Without diabetes (*n* = 4,843)	With prediabetes (*n* = 1,368)	Witt diabetes (*n* = 1,649)
Males	6,150 (44.5%)	3,223 (40.9%)	1,923 (39.7%)	632 (46.2%)	658 (39.9%)
Females	7,658 (55.5%)	4,652 (59.1%)	2,920 (60.3%)	736 (53.8%)	991 (60.1%)
Age	38.0 (30.0-49.0)	39.0 (30.0-50.0)	34.0 (27.0-42.0)	46.0 (37.0-54.3)	53.0 (45.0-60.0)
Glucose (mmol/L)	5.00 (4.60-5.60)	5.00 (4.60-5.60)	4.80 (4.50-5.10)	5.50 (5.00-5.90)	7.10 (5.70-9.20)
C-peptide(nmol/ml)	0.70 (0.52-0.97)	0.66 (0.50-0.88)	0.60 (0.46-0.77)	0.82 (0.64-1.03)	0.78 (0.58-1.04)
Insulin (pmol/L)	69.50 (47.26-108.42)	67.42 (46.56-98.0)	59.08 (42.40-83.40)	85.48 (61.16-119.54)	83.40 (55.60-125.78)
HbA1C (%)	5.40 (5.10-5.80)	5.40 (5.10-5.80)	5.20 (5.00-5.40)	5.8 (5.70-6.00)	6.90 (6.20-8.10)
TAG (mmol/L)	1.10 (0.80-1.60)	1.10 (0.80-1.50)	0.90 (0.70-1.30)	1.20 (0.90-1.60)	1.40 (1.00-1.90)
TC (mmol/L)	4.81 (4.25-5.50)	4.90 (4.30-5.50)	4.80 (4.30-5.40)	5.10 (4.50-5.70)	4.70 (4.00-5.50)
HDL (mmol/L)	1.36 (1.11-1.62)	1.40 (1.15-166)	1.44 (1.20-1.72)	1.30 (1.10-1.55)	1.3 (1.07-1.54)
LDL (mmol/L)	2.90 (2.30-3.50)	2.90 (2.30-3.50)	2.90 (2.30-3.40)	3.1 (2.5-3.7)	2.70 (2.10 - 3.40)
TG/HDL (mg/dL)	1.86 (1.16-3.03)	1.75 (1.13-2.78)	1.46 (0.97-2.29)	2.13 (1.42-3.19)	2.47 (1.64-3.76)
HOMA-IR	2.30 (1.46-3.91)	2.23 (1.47-3.58)	1.81 (1.27-2.62)	2.99 (2.11-4.28)	3.91 (2.40-6.50)
HOMA-B	128.57 (83.83-200.00)	122.86 (81.82-181.43)	134.67 (96.92-192.31)	128.85 (90.91-183.81)	71.46 (39.41-123.43)
HOMA2-IR (ins)	1.31 (0.88-1.92)	1.27 (0.87-1.86)	1.09 (0.79-1.54)	1.62 (1.16-2.25)	1.70 (1.14-2.58)
HOMA2-B (ins)	107.6 (80.80-143.0)	104.30 (79.30-134.50)	110.50 (89.90-138.10)	110.80 (86.58-139.22)	68.10 (40.00-105.30)
HOMA2-IR (c-pep)	1.58 (1.14-2.24)	1.48 (1.10-2.03)	1.30 (0.99-1.69)	1.84 (1.44-2.35)	1.99 (1.43-2.67)
HOMA2-B (c-pep)	122.8 (97.1-154.9)	117.50 (93.65-143.00)	124.2 (104.0-148.1)	121.90 (99.08-146.40)	74.70 (43.20-110.30)
QUICKI	0.337 (0.312-0.360)	0.338 (0.316-0.360)	0.349 (0.330-0.369)	0.324 (0.309-0.341)	0.312 (0.292-0.334)
TyG (mmol/L)	8.43 (8.03-8.88)	8.40 (8.01-8.83)	8.17 (7.87-8.52)	8.61 (8.28-8.88)	9.00 (8.60-9.45)
McAi	7.02 (5.71-8.45)	7.16 (5.98-8.55)	7.82 (6.59-9.03)	6.42 (5.56-7.38)	6.22 (5.21-7.31)
BMI (kg/m^2^)	28.95 (25.41-33.13)	29.15 (25.52-33.27)	27.62 (24.30-31.46)	31.25 (27.79-35.03)	32.36 (28.27-35.61)
Obese**^#^**	5,924 (42.90%)	3,510 (44.57%)	1,630 (33.66%)	833 (60.89%)	1,039 (63.00%)
Overweight**^#^**	4,831 (34.99%)	2,675(33.97%)	1,788 (36.92%)	407 (29.75%)	474 (28.74%)
Normal weight**^#^**	2,777 (20.11%)	1,549 (19.67%)	1,291 (26.66%)	123 (8.99%)	134 (8.13%)
Underweight**^#^**	255 (1.85%)	141 (1.79%)	134 (2.77%)	5 (0.37%)	2 (0.12%)
Family history of diabetes	10,009 (72.49%)	5,748 (72.99%)	3,290 (67.93%)	1,023 (74.78%)	1,423 (86.29%)

Continuous variables are expressed as median (25^th^ -75^th^ percentile), and Mann-Whitney test was used for statistical comparison. Categorical variables are expressed as a number (percentage), and the Chi-square test was used for statistical comparison. A *P*-value < 0.05 was considered statistically significant. ^#^ Participants are classified as persons with obesity if their BMI (≥ 30 kg/m^2^), overweight (24.9 < BMI < 30 kg/m^2^), normal (18.5 ≤ BMI ≤ 24.9kg/m^2^), underweight (BMI < 18.5kg/m^2^), the percentages did not add up to 100 due to missing values. TAG, Triacylglycerol; TC, Total cholesterol; HDL, High Density Lipoprotein; LDL, Low-Density Lipoprotein; HOMA, Homeostasis Model Assessment – IR (insulin resistance)/B(beta cell function); QUICKI, Quantitative Insulin Sensitivity Check Index; McAi, McAuley index for insulin sensitivity. *15 participants were not classified due to missing values.

### 3.2 Correlation between variables and indices of IR

**Figure 2:**
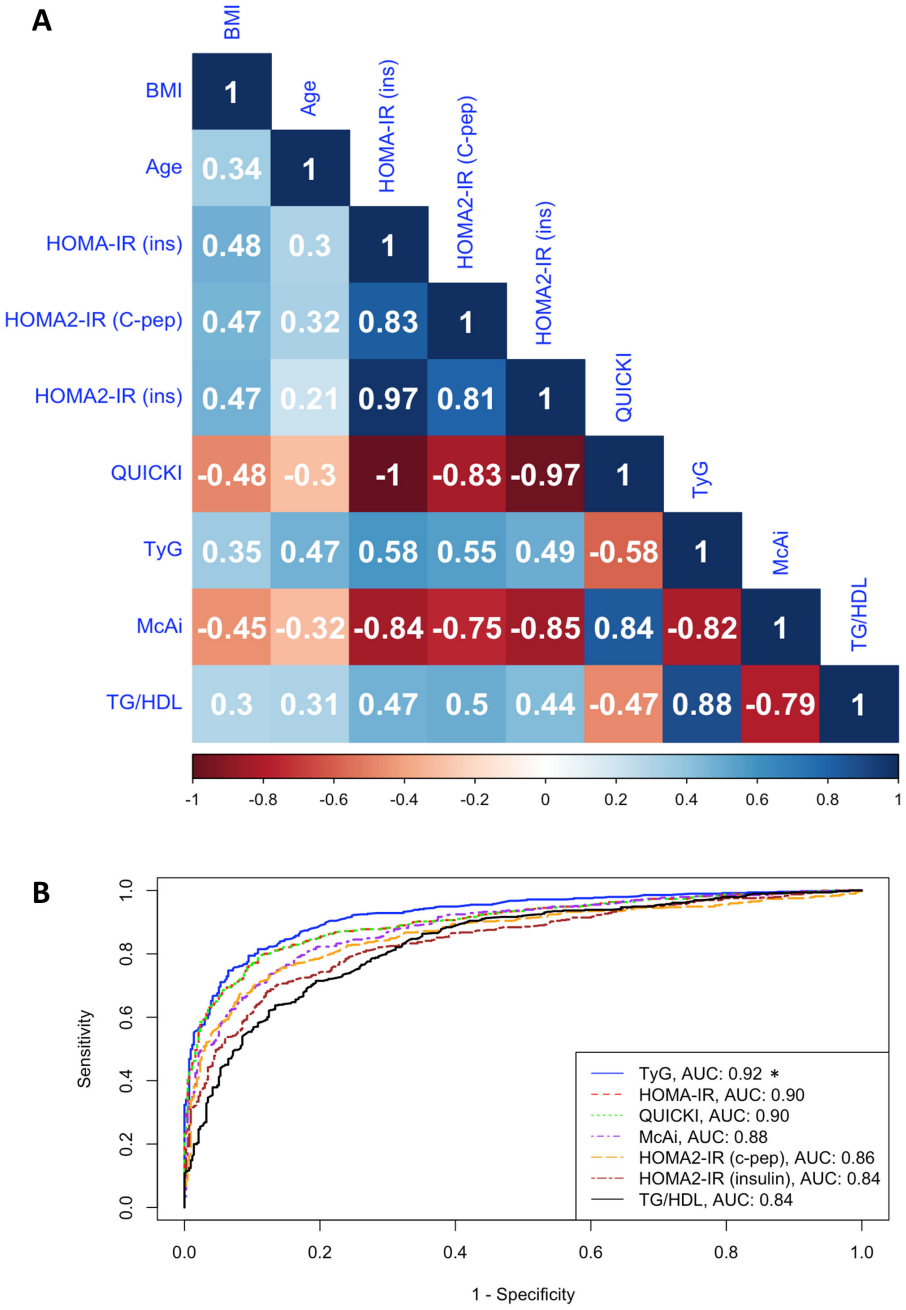
Correlation between IR indices. **(A)**. Heatmap shows the Spearman correlation coefficient (ρ) of age, BMI, and IR indices in the fasting cohort (*n* = 7,875). HOMA-IR (ins), HOMA2-IR (C-pep), HOMA2-IR (insulin), TyG, and TG/HDL assess insulin resistance, while QUICKI and McAi assess insulin sensitivity. **(B)**. ROC-curve analysis shows the discriminatory performances (AUC) of indices of IR in the stratification of IR and IS individuals in the testing cohort. *P < 0.05 based on DeLong’s pairwise test comparing the AUC of TyG to other indices.

The results of Spearman correlation coefficient (ρ) between age, BMI, and indices of IR are presented in **Figure 2A**. BMI showed moderate correlation with age (ρ = 0.34) and IR indices (ρ = 0.30 to 0.48 and -0.45 to -0.48), while age also moderately correlated with IR indices (ρ = 0.21 to 0.47 and -0.30 to -0.32). IR indices showed maximal correlation (ρ = -1.00) between HOMA-IR and QUICKI and moderate correlation (ρ = 0.44) between HOMA2-IR (insulin) and TG/HDL. The observed negative correlation of QUICKI and McAi with BMI and other indices is unsurprising since these indices gauge insulin sensitivity rather than IR (**Figure 2A**).

### 3.3 Optimal cut-offs for indices of IR

**Table 2** presents the identified cut-offs for indices of IR determined using the discovery dataset, with the corresponding sensitivity, specificity, and AUC in the combined and gender-specific analyses in the testing dataset. The performance metrics were based on the indices’ discriminative and diagnostic capacity between apparently IS and IR individuals in the testing dataset as described in the methods section. The characteristics of the IS and IR participants are summarized in **Supplementary Table S1**.

**Table 2:** Cut-off points and performance metrics for insulin resistance indices.

Index	Gender	Cut-off *	Sensitivity	Specificity	AUC (95% CI)
HOMA-IR	All	≥1.878	0.87	0.77	0.90 (0.88-0.92)
HOMA-IR	Male	≥1.711	0.92	0.71	0.93 (0.89-0.95)
HOMA-IR	Female	≥1.984	0.82	0.81	0.88 (0.86-0.91)
HOMA2-IR (insulin)	All	≥1.128	0.77	0.77	0.84 (0.82-0.87)
HOMA2-IR (insulin)	Male	≥1.036	0.85	0.72	0.87 (0.83-0.91)
HOMA2-IR (insulin)	Female	≥1.177	0.72	0.79	0.83 (0.80-0.86)
HOMA2-IR (C-peptide)	All	≥1.307	0.83	0.76	0.86 (0.84-0.88)
HOMA2-IR (C-peptide)	Male	≥1.264	0.88	0.76	0.89 (0.85-0.92)
HOMA2-IR (C-peptide)	Female	≥1.320	0.78	0.76	0.84 (0.80-0.88)
QUICKI	All	≤0.347	0.87	0.77	0.90 (0.88-0.92)
QUICKI	Male	≤0.352	0.92	0.71	0.93 (0.90-0.95)
QUICKI	Female	≤0.344	0.82	0.81	0.88 (0.86-0.91)
TyG	All	≥8.281	0.90	0.79	0.92 (0.91-0.94)
TyG	Male	≥8.416	0.90	0.80	0.93 (0.91-0.96)
TyG	Female	≥8.168	0.91	0.74	0.92 (0.91-0.94)
McAi	All	≤7.727	0.82	0.78	0.88 (0.86-0.91)
McAi	Male	≤7.556	0.83	0.81	0.89 (0.85-0.92)
McAi	Female	≤7.892	0.84	0.73	0.88 (0.85-0.91)
TG/HDL	All	≥1.718	0.71	0.81	0.84 (0.81-0.87)
TG/HDL	Male	≥2.189	0.72	0.80	0.83 (0.78-0.86)
TG/HDL	Female	≥1.317	0.82	0.72	0.84 (0.81-0.88)

HOMA-IR, Homeostasis Model Assessment – Insulin Resistance; QUICKI, Quantitative Insulin Sensitivity Check Index; TyG, Triglyceride-Glucose index; McAi, McAuley index; TG/HDL, Triglyceride/High Density Lipoprotein ratio. *Cut-off derived from the discovery dataset. Performance metrics (Sensitivity, Specificity, and AUC) were derived from the testing dataset.

All indices demonstrated strong discriminatory capacity between IR and IS participants, with AUC values ranging from 0.83 (0.78 – 0.86 95% CI) to 0.92 (0.91 – 0.94 95% CI) (**Figure 2B**; **Table 2**). TyG consistently outperformed all other indices, followed by HOMA-IR and QUICKI. For the combined and female-specific analysis, TyG demonstrated the best discriminative and diagnostic performance (cut-off ≥ 8.281, AUC: 0.92, sensitivity: 0.90, specificity: 0.79) and (cut-off ≥ 8.168, AUC: 0.92, sensitivity: 0.91, specificity: 0.74) respectively. However, for male-specific analysis, TyG, HOMA-IR, and QUICKI, demonstrated equal discriminative capacity (AUC: 0.93), but TyG showed better diagnostic value (cut-off ≥ 8.416, AUC: 0.93, sensitivity: 0.90, specificity: 0.80). Similarly, the TyG index demonstrated the best overall diagnostic performance, with a positive likelihood ratio of 4.29 and a negative likelihood ratio of 0.13, outperforming all other evaluated indices (**Supplementary Table S2**). Of note, TyG consistently remained the top-performing index in analysis restricted to participants with newly diagnosed diabetes who were not on any diabetes medication, thereby eliminating the potential influence of treatment on our results (**Supplementary Table S3**).

In addition, we conducted exploratory analyses to investigate the effects of using the ROC-curve analysis for cut-off identification and assessment as adopted by some previous studies (25). We performed ROC-curve analyses in the discovery dataset and identified the cut-offs that were tested in the testing dataset (**Supplementary Table S4**). Although the cut-offs identified using the ROC-curve analysis provided better performance metrics, considering our IR criteria, cut-offs identified through this method were subject to overfitting. Therefore, for downstream analyses, we adopted the 75th percentile approach as shown in **Table 2**, in line with recommendations of the World Health Organization (WHO) (26).

### 3.4 Prevalence of insulin resistance

**Figure 3:**
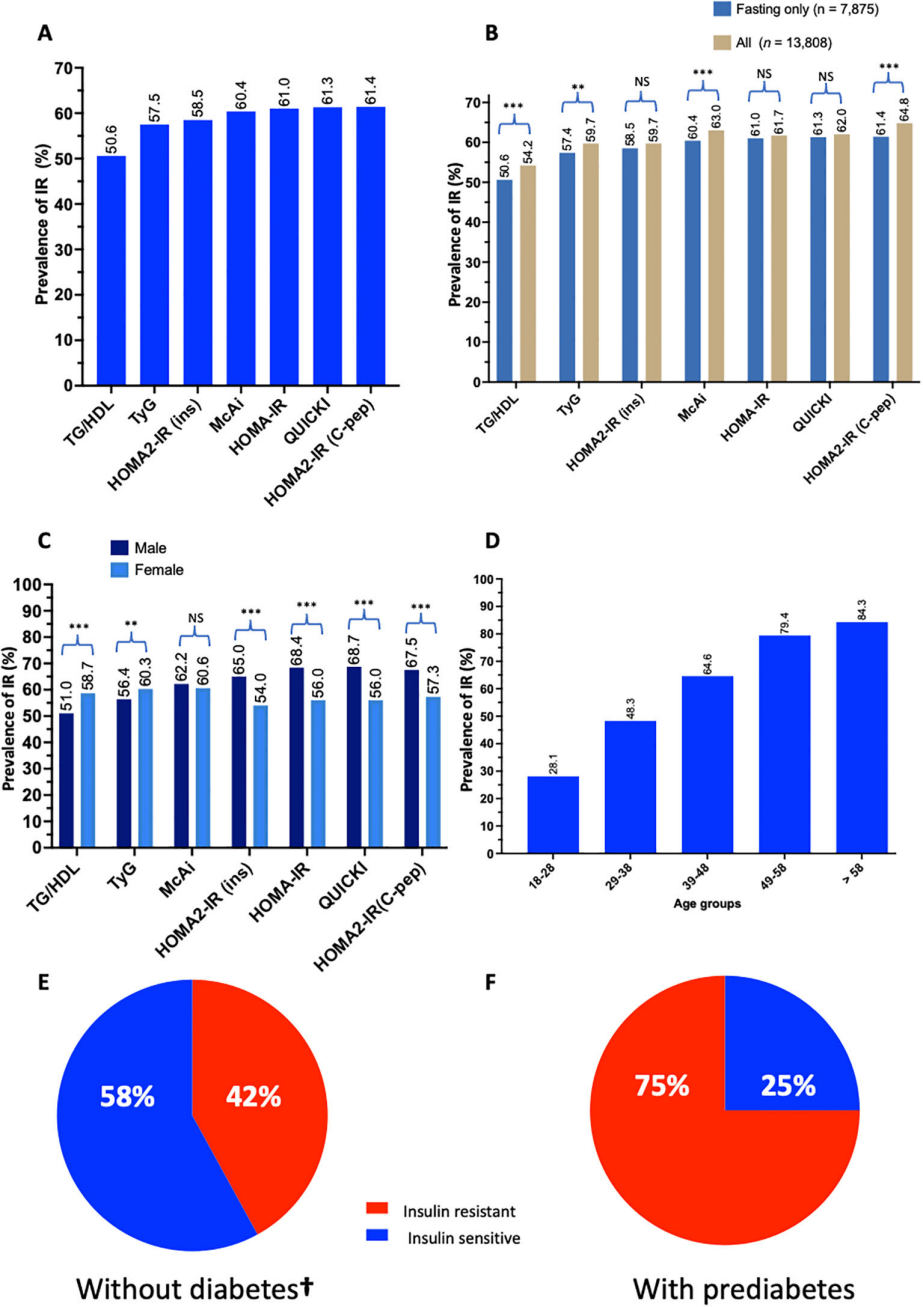
Prevalence of IR in Qatar. Bar charts represent **(A)** the prevalence of IR in QBB individuals (*n* = 7,785) with fasting measurements using different IR indices, **(B)** the differences in determining the prevalence of IR in QBB individuals using IR indices in fasting (*n* = 7,785) and all QBB participants (*n* = 13,808), **(C)** the gender-wise prevalence of IR using gender-specific cut-offs for different IR indices **(D)** the prevalence of IR in different age groups using TyG index: 18-28 (*n* = 1,627), 29-38 (*n* = 2,162), 39-48 (*n* = 1,796), 49-58 (*n* = 1,423) and > 58 (*n* = 867) years. Pie chart represents the proportion of participants **(E)** without diabetes (*n* = 4,843) and **(F)** with prediabetes (*n* = 1,368) that are IR as identified by the TyG index. TG/HDL: Triglyceride/High-density lipoprotein ratio, TyG: Triglyceride-Glucose index, McAi: McAuley index, HOMA: Homeostatis Model Assessment, IR: Insulin Resistance, C-pep: C-peptide, ins: insulin. **: chi-square test p-value < 0.005, *** < 0.0005, NS, Not significant. †, This group included only individuals with normal fasting glucose and normal HbA1C without prediabetes. **(A–F)** are based on fasting measurement.

The prevalence of IR in the QBB cohort varied depending on the index used, ranging from 51% by TG/HDL to 65% by HOMA2-IR (C-peptide) (**Figures 3A, B**). Some indices were more sensitive to fasting measurements than others. The overall prevalence was statistically different when fasting duration was not considered in TG/HDL (p-value = 5.6 x 10^-7^), TyG (p-value = 1.4 x 10^-3^), McAuley (*P*-value = 1.7 x 10^-4^) and HOMA2-IR (C-peptide) (*P* -value = 8.0 x 10^-7^) but not significant in HOMA2-IR (insulin) (*P*-value = 8.4 x 10^-2^), HOMA-IR (*P*-value = 2.7 x 10^-1^) and QUICKI (*P*-value = 3.0 x 10^-1^) (**Figure 3B**). The prevalence of IR was higher in males by most indices except TG/HDL and TyG, where IR was more prevalent in females (**Figure 3C**). Moreover, there was a significant difference in the prevalence of IR between males and females in all the indices (*P*-value < 5 x 10^-10^), except McAuley (*P*-value = 1.5 x 10^-1^). Since TyG demonstrated the best performance, we used this metric to estimate the prevalence of IR in age groups as well as in in participants without diabetes and those with prediabetes. The prevalence of IR increases with age (chi-square *p* < 2.2 x 10^-16^), with the lowest prevalence (28.1%) in 18–28 years and the highest (84.3%) in > 58 years (**Figure 3D**). The prevalence of IR among individuals without diabetes was estimated at 42% and 75% among persons with prediabetes (**Figures 3E, F**). Individuals without diabetes but with insulin resistance (IR) exhibited elevated fasting insulin, fasting C-peptide, HbA1C, BMI and tend to be older compared to their insulin-sensitive (IS) counterparts (**Supplementary Table S5**).

### 3.5 Concordance of IR indices

We investigated the concordance of different IR indices in their diagnostic accuracy for IR. Combined, the proportion of individuals identified as IR by at least 1, 2, 3, 4, 5, 6, or 7 indices corresponds to 6,254 (79.4%), 5,742 (72.9%), 5,364 (68.1%), 4,817 (61.2%), 4,166 (52.9%), 3,422 (43.5%), and 2,582 (32.8%) respectively (**Supplementary Figure S1A**). Some indices identified certain individuals as IR that no other index could identify. For instance, HOMA2-IR (C-peptide) exclusively identified 208 individuals as IR that could not be identified by any other index. Similarly, TyG and TG/HDL alone identified 148 and 120 individuals as IR, respectively, not identified by all other indices (**Supplementary Figure S1B**).

## 4 Discussion

HOMA-IR is among the widely used indirect measures of IR (11). The cut-off identified herein for HOMA-IR (≥ 1.878, sensitivity: 87%, specificity: 77%, AUC: 0.90) is higher than the previously reported threshold of >1.6 reported in Oman (27) but lower than reports from Hungary (> 2.32) (21), Turkey (> 2.46) (28), Brazil (> 2.7) (29) and Korea (> 3.04) (30). HOMA2 is suggested to be a more accurate marker for IR as it better reflects the metabolic processes and accommodates modern insulin assays (12) However, limited studies reported the reference cut-offs for HOMA2 and mostly relied on insulin measurements instead of C-peptide, which is more accurate and less affected by exogenous insulin and medications (31). We reported a HOMA2-IR cut-off of ≥ 1.128 (sensitivity: 77%, specificity: 77%, AUC: 0.84) and ≥ 1.307 (sensitivity: 83%, specificity: 76%, AUC: 0.86) using insulin and C-peptide, respectively. Our identified cut-off for HOMA2-IR is lower than previous reports from Kuwait (> 1.4) (32), Turkey (> 1.4) (28) and Brazil (> 1.8) (29). We observed that the variability in HOMA-1 cut-offs between countries is less pronounced with HOMA2. In addition, HOMA-1 cut-offs identified in this study were consistently higher than HOMA-2 cut-offs, a finding that is in agreement with previous studies (29, 33).

QUICKI is a logarithmic variation of HOMA-1 (HOMA-IR) that strongly correlates with HEC (13). We proposed a cut-off of ≤ 0.347 (sensitivity: 87%, specificity: 77%, AUC: 0.90) as the optimum diagnostic threshold for IR. The proposed cut-off was higher in our study than in studies from Hungary (< 0.336) (21), Korea (< 0.32) (30) and India (< 0.32) (20). Notably, the similar diagnostic and discriminative performances of QUICKI and HOMA-1 in our study suggest the redundancy of concurrent use of both indices. Moreover, the approximate cut-off for QUICKI should be used with caution. QUICKI is a logarithmic transformation shrunk to a narrow range and rough approximation could result in substantially erroneous results. Similarly, the cut-off identified for McAi in this study (≤ 7.272, sensitivity: 82%, specificity: 78%, AUC: 0.88) is higher than what was reported from Hungary (< 5.989) (21) and India (< 6.05) (20). The TG/HDL ratio is essential in identifying IR and persons with dyslipidemia, who consequently are at high risk of CVD (14). The cut-off reported here (≥ 1.718, sensitivity: 71%, specificity: 81%, AUC: 0.84) is lower than what was reported in the original TG/HDL study (≥ 3.5) (14), non-Hispanic whites and Mexican Americans (≥ 3.0) and non-Hispanic blacks (≥ 2.0) (34) but higher than report from Hungary (≥ 1.274) (21).

TyG is another surrogate marker for IR developed by Simental et al. (2008) as a product of fasting glucose and triglycerides (15). TyG has been shown to strongly correlate with HEC and achieved up to 96% sensitivity for IR using HEC as a benchmark (35). Mechanistically, the TyG index captures the combined metabolic disturbances in glucose and lipid metabolism that are central to insulin resistance. A hallmark of insulin resistance is compensatory hyperinsulinemia, which promotes lipolysis in adipose tissue and elevates circulating free fatty acid (FFA) levels and lipid intermediates (36). These components directly interfere with insulin signaling pathways at the receptor level and their intracellular effectors, such as IRS-1 (insulin receptor substrate-1) and PI3K (phosphatidylinositol-3-kinase) (37). This is further aggravated by imbalances in adipokines, such as adiponectin and leptin particularly in obesity (38). The excess FFAs are taken up by the liver and re-esterified, resulting in increased hepatic lipogenesis, increasing the production and circulating levels of triglycerides (39). Concurrently, hepatic insulin resistance reduces glycogenesis and enhances gluconeogenesis, contributing to elevated fasting glucose levels (40). The simultaneous elevation of triglycerides and glucose in insulin resistance underpins the physiological rationale for using the TyG index as a surrogate marker of insulin resistance. In this study, we identified TyG cut-off of ≥ 8.281 with a sensitivity of 90%, specificity of 79%, and AUC of 0.92. This cut-off was lower than previous reports from India (≥ 9.88) (20) but higher than reports from Hungary (≥ 4.694). Our study indicated TyG has a better diagnostic performance for IR, surpassing all other analyzed indices, including the widely used HOMA-IR. This finding is in agreement with findings from China (41) Poland (18), Korea (42) and Europe (43). One of the main advantages of TyG is the feasibility of glucose and triglyceride measurements across clinical laboratories. Moreover, TyG can be utilized irrespective of individuals’ insulin treatment status. Recently, a large multicontinental longitudinal study comprising of cohorts from 22 countries reported that TyG is associated with incidence of CVD, cardiovascular mortality, and T2D (44), further emphasizing the role of IR in the pathogenesis of these diseases. Of note, other IR-related indices calculated as a product of biochemical and anthropometric measurements such as TyG-body mass index (TyG-BMI), TyG-waist circumference (TyG-WC) and Single Point Insulin Sensitivity Estimator (SPISE) were shown with promising results in previous studies (45, 46). However, the present study was restricted to clinically useful indices calculated from biochemical measurements only. Overall, our data suggests that TyG has the potential to be used in the Middle Eastern population for IR screening with appreciable accuracy. Nonetheless, given the criteria and scope of our study, we acknowledge that the TyG index may not necessarily outperform other indices in all disease contexts, particularly those with distinct pathophysiological mechanisms.

The prevalence of IR varies widely across countries. The prevalence of IR is low in Europeans; ~15.5% in Danish (47) and 17.5% in France (48), and higher in other countries, including Thailand (23.3%) (49), Turkey (33.2%) (28), US-Texas (39.1%) (50), Lebanon (44.6%) (51), Venezuela (46.5%) (52) and Iran (51%) (53). We estimated the overall prevalence of IR in Qatar in the range between 51-65% and ~42% among persons without diabetes, which puts the prevalence of IR in Qatar among the highest in the region and globally. The high prevalence is supported by the finding that 34% of all the study participants were unanimously identified as IR by all the indices analyzed. Previous studies also reported a prevalence of 16% among young Omani students (54) and 7-37% among non-obese healthy young Qatari females (4). The high prevalence of IR in the Qatari population could be attributed to the commensurate rise in obesity, as evidenced by the results of our study, where more than 33% of individuals without diabetes were obese, and more than 36% were overweight. This could be a consequence of the transition from active to sedentary lifestyle observed in the region (4). However, the prevalence of IR reported across countries should be interpreted cautiously as factors such as the cut-off, study design, cohort, and analytical method may inflate or deflate estimates. Studies have shown that IR can predict up to 40% risk of T2D in individuals with obesity and up to 80% in individuals without obesity (55), suggesting that about 42% of QBB participants without diabetes may develop T2D in the future. The higher prevalence of IR in men compared to women could be explained by the protective effect of estrogen in women against IR (56). Our findings on gender disparity in the prevalence of IR agree with reports from France, where an IR prevalence of 23% was reported in men compared to 12% in women (48).

Overall, our pioneering study leveraged the deeply characterized data of QBB participants (*n* = 7,875) to propose the combined and gender-specific cut-offs for the seven commonly used and clinically useful indices of IR. We showed that all studied markers have good predictive performances for IR. TyG emerged as the most accurate, robust, and accessible measure of IR in the Qatari population. Our findings strongly support its recommended use for IR assessment and prediction of metabolic disorders. However, our findings could not be validated using the gold standard method of IR detection via HEC due to data unavailability. Conducting HEC in a large cohort is cost prohibitive. Also, we acknowledge that the results reported here may not be generalizable to other populations, and population-specific studies are warranted. Overall, our study is the largest of its kind conducted in the Middle East, and proposed cut-offs that could serve as a reference in the region for IR screening. The alarmingly high prevalence of IR in the population underscores the need to prioritize early screening for IR to mitigate the onset of IR-associated diseases, including T2D and CVD.

## Data Availability

The data analyzed in this study is subject to the following licenses/restrictions: The data analyzed in this study are subject to the following licenses/restrictions: Access to QBB phenotype data can be obtained through an ISO-certified protocol, which involves submitting a project request subject to approval by the Institutional Review Board of the QBB. Requests to access these datasets should be directed to https://www.qphi.org.qa/research/how-to-apply.
