# Supplementary material for: Evaluating indices of insulin resistance and estimating the prevalence of insulin resistance in a large biobank cohort
Source: Front Endocrinol (Lausanne). 2025 May 12;16:1591677. doi: 10.3389/fendo.2025.1591677 (PMC12104043; doi:10.3389/fendo.2025.1591677)
Supplement: Supplementary file 1 [file DataSheet1.pdf]

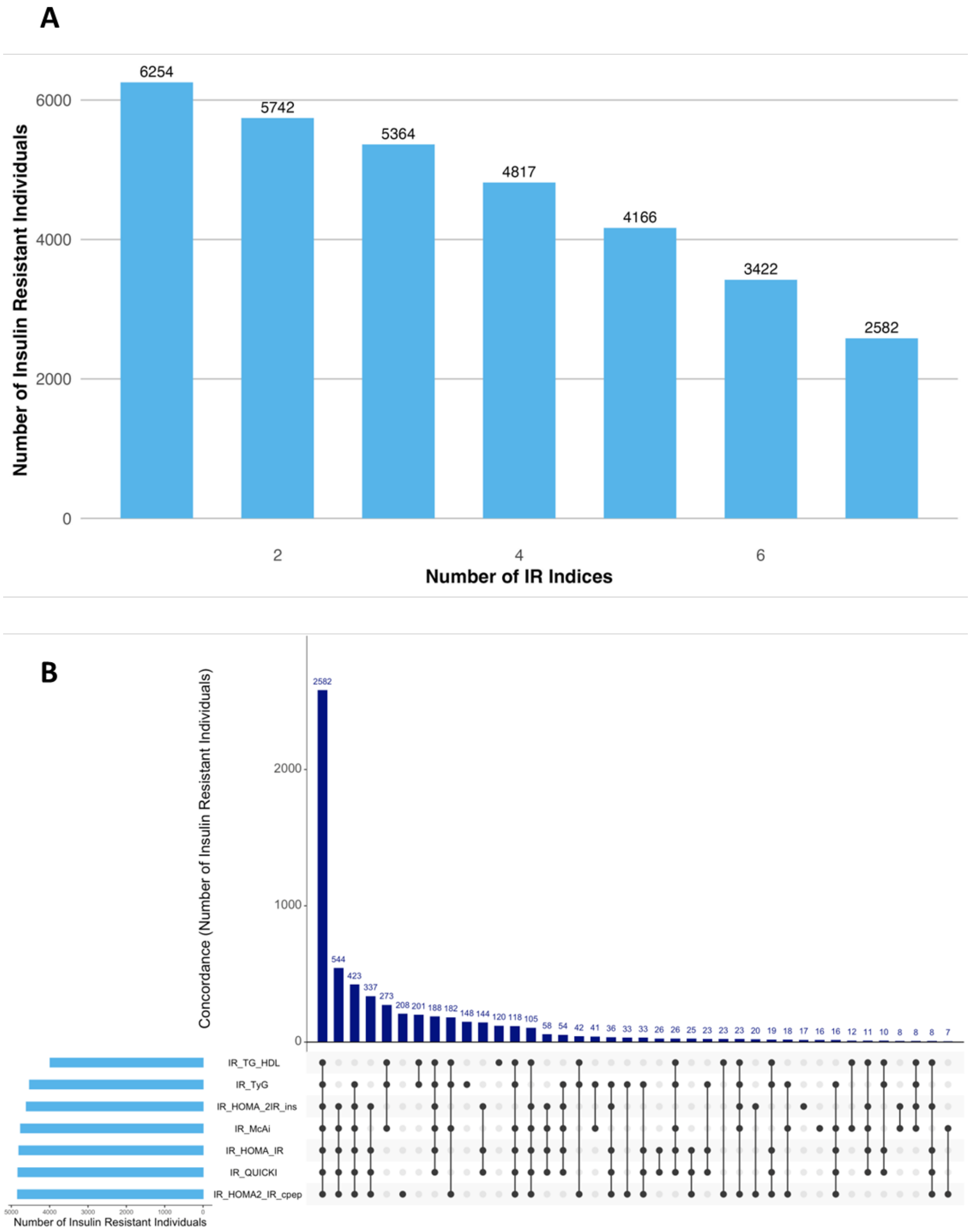

Supplementary Figure 1. Concordance of IR indices. (A) The plot illustrates the number of individuals identified as IR by at least 1 to 7 indices. X-axis represents the number of indices, ranging from 1 to 7, while the y-axis shows the number of individuals identified by each corresponding number of indices. Bars represents the cumulative number of individuals identified as IR by at least the specified number of indices. **(B)** UpSetR plot shows the concordance of IR indices in identifying IR individuals. Bar plot shows the concordance of IR indices in identifying IR individuals. Each bar corresponds to the number of individuals in the concordance among indices (dots connected by lines). The bottom horizontal bars (rows) represent the total number of IR individuals identified by each index as IR.

Supplementary Table 1. Characteristics of IS and IR participants.

Parameter	Insulin Sensitive (IS)		Insulin Resistant (IR)	
	Discovery (n = 960)	Testing (n = 433)	Discovery (n = 1,158)	Testing (n = 491)
Age	28.0 (24.0-35.0)	28.0 (23.0-35.0)	53.0 (45.0-60.0)	53.0 (45.0-60.0)
Male	396 (41.2%)	147 (33.9%)	458 (39.6%)	200 (59.3%)
Female	564 (58.8%)	286 (66.1%)	700 (60.4%)	291 (40.7%)
BMI	22.4 (20.6-23.8)	22.6 (20.8-23.8)	31.6 (28.1-35.6)	32.1 (28.7-35.8)
HOMA-IR	1.38 (1.04-1.88)	1.41 (1.06-1.82)	3.87 (2.36-6.34)	4.02 (2.47-6.90)
HOMA2-IR (C-peptide)	1.05 (0.84-1.31)	1.02 (0.84-1.29)	1.99 (1.42-2.64)	1.99 (1.47-2.74)
HOMA2-IR (insulin)	0.85 (0.65-1.13)	0.87 (0.66-1.10)	1.69 (1.13-2.55)	1.76 (1.16-2.70)
QUICKI	0.364 (0.347-0.380)	0.362 (0.349-0.380)	0.313 (0.293-0.335)	0.311 (0.290-0.333)
TyG	7.98 (7.72-8.28)	7.94 (7.70-8.21)	9.0 (8.60-9.46)	8.99 (8.61-9.43)
McAi	8.77 (7.73-9.98)	8.77 (7.88-10.09)	6.21 (5.21-7.35)	6.22 (5.23-7.20)
TG/HDL	1.09 (0.80-1.72)	1.08 (0.80-1.58)	2.47 (1.65-3.78)	2.47 (1.57-3.65)

HOMA-IR: Homeostasis Model Assessment – Insulin Resistance, QUICKI: Quantitative Insulin Sensitivity Check Index, TyG: Triglyceride-Glucose index, McAi: McAuley index, TG/HDL: Triglyceride/High Density Lipoprotein ratio.

Supplementary Table 2: Likelihood ratio for Indices of Insulin Resistance.

Index	Sensitivity	Specificity	LR+	LR-
HOMA-IR	0.87	0.77	3.78	0.17
HOMA2-IR (insulin)	0.77	0.77	3.35	0.30
HOMA2-IR (C-peptide)	0.83	0.76	3.46	0.22
QUICKI	0.87	0.77	3.78	0.17
TyG	0.9	0.79	4.29	0.13
McAi	0.82	0.78	3.73	0.23
TG/HDL	0.71	0.81	3.74	0.36

LR+: Positive Likelihood Ratio, LR-: Negative Likelihood Ratio

Supplementary Table 3: Cut-off points and performance metrics for insulin resistance indices using only newly diagnosed T2D cases (n = 149) as insulin resistant.

Index	Cut-off*	Sensitivity	Specificity	AUC (95% CI)
HOMA-IR	≥1.878	0.94	0.77	0.94 (0.92-0.96)
HOMA2-IR (insulin)	≥1.128	0.87	0.77	0.89 (0.86-0.92)
HOMA2-IR (C-peptide)	≥1.307	0.94	0.76	0.94 (0.94-0.96)
QUICKI	≤0.347	0.94	0.77	0.94 (0.92-0.96)
TyG	≥8.281	0.95	0.79	0.96 (0.95-0.98)
McAi	≤ 7.727	0.91	0.78	0.93 (0.91-0.95)
TG/HDL	≥ 1.718	0.83	0.81	0.89 (0.86-0.92)

HOMA-IR: Homeostasis Model Assessment – Insulin Resistance, QUICKI: Quantitative Insulin Sensitivity Check Index, TyG: Triglyceride-Glucose index, McAi: McAuley index, TG/HDL: Triglyceride/High Density Lipoprotein ratio. * cut-off derived from the discovery dataset. Performance metrics (Sensitivity, Specificity, and AUC) were derived using only newly diagnosed T2D as insulin resistant (n = 149).

Supplementary Table 4. Results for receiver-operator characteristics curve analysis for Indices of Insulin resistance.

Index	Gender	Cut-off	Percentile*	Discovery (70%)			Testing (30%)		
				Sensitivity	Specificity	AUC	Sensitivity	Specificity	AUC
HOMA-IR	All	≥ 2.209	85.63	0.78	0.86	0.89	0.80	0.87	0.90
HOMA-IR	Male	≥ 2.111	86.11	0.82	0.86	0.92	0.87	0.85	0.93
HOMA-IR	Female	≥ 2.335	85.82	0.76	0.86	0.87	0.74	0.89	0.88
HOM2-IR (insulin)	All	≥ 1.130	75.42	0.75	0.75	0.82	0.77	0.77	0.84
HOM2-IR (insulin)	Male	≥ 1.117	80.81	0.74	0.81	0.84	0.81	0.76	0.87
HOM2-IR (insulin)	Female	≥ 1.272	80.50	0.69	0.81	0.81	0.69	0.85	0.83
HOM2-IR (C-peptide)	All	≥ 1.431	83.65	0.75	0.84	0.85	0.76	0.85	0.86
HOM2-IR (C-peptide))	Male	≥ 1.421	83.59	0.79	0.84	0.86	0.85	0.79	0.89
HOM2-IR (C-peptide))	Female	≥ 1.580	90.60	0.65	0.91	0.84	0.64	0.92	0.84
QUICKI	All	≤ 0.338	85.94	0.78	0.86	0.89	0.80	0.88	0.90
QUICKI	Male	≤ 0.341	85.61	0.82	0.86	0.91	0.87	0.85	0.93
QUICKI	Female	≤ 0.336	85.46	0.76	0.86	0.87	0.74	0.89	0.88
TyG	All	≥ 8.462	86.56	0.82	0.87	0.91	0.81	0.89	0.92
TyG	Male	≥ 8.622	87.12	0.79	0.87	0.90	0.81	0.89	0.93
TyG	Female	≥ 8.44	91.67	0.82	0.92	0.93	0.77	0.91	0.92
McAi	All	≤ 7.461	81.15	0.77	0.81	0.86	0.79	0.83	0.88
McAi	Male	≤ 7.132	85.10	0.73	0.85	0.84	0.77	0.86	0.89
McAi	Female	≤ 7.447	82.98	0.77	0.83	0.86	0.78	0.84	0.88
TG/HDL	All	≥ 1.478	68.96	0.81	0.69	0.82	0.79	0.71	0.84
TG/HDL	Male	≥ 1.963	69.95	0.75	0.70	0.79	0.78	0.75	0.83
TG/HDL	Female	≥ 1.347	76.24	0.82	0.76	0.86	0.80	0.73	0.84

HOMA-IR: Homeostasis Model Assessment – Insulin Resistance, QUICKI: Quantitative Insulin Sensitivity Check Index, TyG: Triglyceride-Glucose index, McAi: McAuley index, TG/HDL: Triglyceride/High Density Lipoprotein ratio. *Percentile of the cut-off in insulin sensitive individuals in the discovery cohort.

Supplementary Table 5. Characteristics of insulin sensitive and insulin resistant among individuals without diabetes

Parameter	Insulin sensitive (*n* = 2831)	Insulin resistant (*n* = 2012)	*P*-value
Age	31.0 (25.0-39.0)	37.0 (31.0-46.0)	5.6×10^{-73}
Glucose (mmol/L)	4.7 (4.4-5.0)	5.0 (4.7-5.3)	2.2×10^{-90}
HbA1C	5.2 (5.0-5.4)	5.3 (5.1-5.5)	2.0×10^{-33}
C-peptide(nmol/ml)	0.53 (0.42-0.66)	0.71 (0.56-0.92)	5.0×10^{-171}
Insulin (pmol/L)	50.73 (38.23-70.19)	72.28 (53.52-100.78)	4.3×10^{-127}
BMI	26.48 (23.18-30.19)	29.14 (25.97-33.01)	1.3×10^{-66}
